# Substrate stiffness modulates collective colony expansion of the social bacterium *Myxococcus xanthus*

**DOI:** 10.1063/5.0226619

**Published:** 2025-01-17

**Authors:** Nuzhat Faiza, Roy Welch, Alison Patteson

**Affiliations:** 1 Physics Department, Syracuse University, Syracuse, New York 13244, USA; 2 BioInspired Institute, Syracuse University, Syracuse, New York 13244, USA; 3 Biology Department, Syracuse University, Syracuse, New York 13244, USA

## Abstract

Many cellular functions depend on the physical properties of the cell's environment. Many bacteria have different types of surface appendages to enable adhesion and motion on various surfaces. *Myxococcus xanthus* is a social soil bacterium with two distinctly regulated modes of surface motility, termed the social motility mode, driven by type IV pili, and the adventurous motility mode, based on focal adhesion complexes. How bacteria sense different surfaces and subsequently coordinate their collective motion remains largely unclear. Using polyacrylamide hydrogels of tunable stiffness, we found that wild type *M. xanthus* spreads faster on stiffer substrates. Here, we show that using motility mutants that disrupt adventurous motility suppresses this substrate stiffness response, suggesting focal adhesion-based adventurous motility is substrate stiffness dependent. We also show that modifying surface adhesion by adding adhesive ligands, chitosan, increases the amount of *M. xanthus* flairs, a characteristic feature of adventurous motility. Taken together, we hypothesize a central role of *M. xanthus* adventurous motility as a driving mechanism for surface and surface stiffness sensing.

## INTRODUCTION

Most bacteria live in surface-dwelling multicellular colonies. Bacteria surface motility plays an important role in various biological and ecological settings, such as microbial infections, the fouling of water systems, and the carbon equilibrium within different ecosystems. Understanding the role of the surface environment in mediating and influencing the motility of microorganisms is, therefore, central to addressing a wide range of scientific and engineering challenges. The mechanical stiffness of a surface can strongly affect microbe motility, and microbes have evolved various specialized machinery to recognize different surfaces and promote surface exploration. For example, type IV pili are highly conserved appendages that drive propulsion by cyclic extension, attachment, and retraction. The tension on the pili is mediated by surface stiffness, which tunes the cell's transcriptional response to different surfaces in *Pseudomonas aeruginosa.*^1^ In mammalian cells, the field of mechanobiology initially emerged to explore how cells react to physical changes in their surroundings.^2–4^ Despite the intriguing new data that suggest bacteria can also sense and respond to physical cues of their environment,^2,5–11^ our understanding of the molecular mechanisms is largely incomplete.

**FIG. 1.**
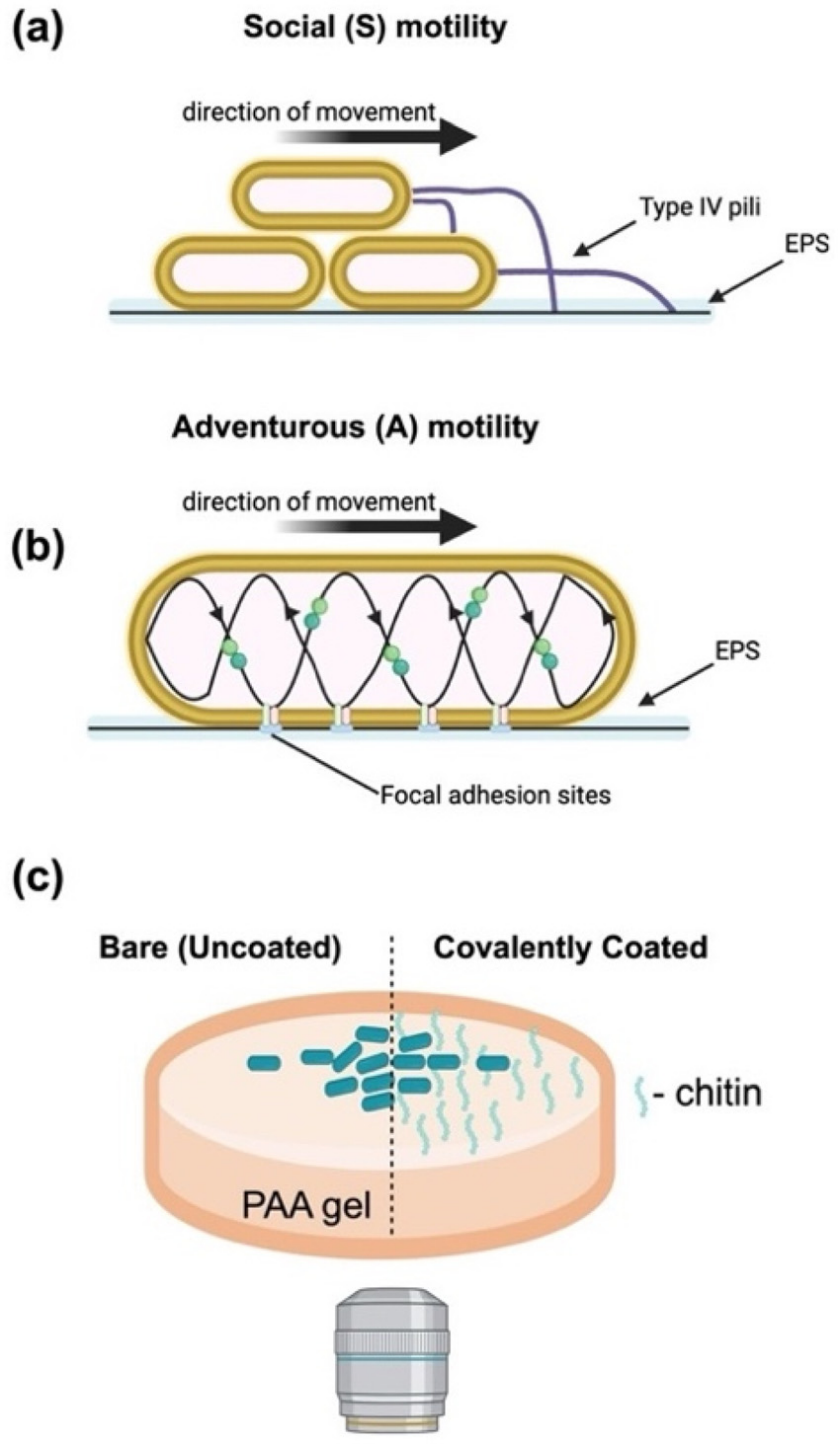
Schematic of the experimental study. *M. xanthus* adheres and moves to surfaces using two distinct motilities. (a) Social (S) involved attachment and retraction of a surface appendage, type IV pili, that adheres to other cells and cell-secreted EPS trails. (b) Adventurous (A) motility involves secretion of an EPS slime trail and formation of bacterial focal adhesion sites that couple to helically trafficked motor. (c) In this study, *M. xanthus* colonies are cultured and imaged on polyacrylamide (PAA) gels of tunable stiffness and surface conditions. Two experimental surface conditions are used: (i) inert uncoated gels and (ii) covalently linked chitosan on the surface.

In this study, we used *M. xanthus* as a model organism of bacterial collective surface motility. Many microbes have evolved rudimentary forms of multicellularity and collective behaviors but, myxobacteria, which belong to the phylum *Myxococcota*,^12^ display remarkably sophisticated forms of collective behavior, including swarming, predation, slime trail creation, and multicellular fruiting body development.^13–16^ *M. xanthus* possesses two genetically distinct motility systems [Figs. 1(a) and 1(b)].^17,18^ The first is called social (S)-motility and is powered by the retraction of type IV pili.^19^ The second is adventurous (A)-motility, commonly called gliding, which is powered by an inner membrane motor that applies force to the substrate at adhesions.^20–22^ The two motility systems of *M. xanthus* show different selective advantages on various surfaces. A pioneering study in the field showed that, when grown on soft agar substrates, *M. xanthus* primarily uses S-motility but on hard agar *M. xanthus* primarily uses A-motility modes.^23^ Surface sensing studies of *M. xanthus* are mostly conducted on agar surfaces like many other bacteria species. Recent studies have shown that gliding motility-based cell speeds are largely constant with agar concentration but accelerate for sufficiently large agar concentrations (>6% agar).^24^ These observations suggest that *M. xanthus* adapt and coordinate their motility modes based on the physical features of their environment, but the main mechanisms are not yet lucid.

To determine how bacteria respond to physical changes in their substrate, our group and others have been employing synthetic polyacrylamide (PAA) hydrogels, which have become a standard for mechanobiology studies in mammalian cells^25–27^ and which have several advantages for testing models of bacteria–substrate interactions compared to commonly used agar surfaces.^1,28–30^ Agar is isolated from marine algae and its isolation process makes it difficult to define and reproduce its chemical properties.^31,32^ It is also difficult to control and tune the physical properties of agar. For example, increasing agar concentration not only increases substrate stiffness but also decreases the agar pore size and the ability of nutrients to diffuse into the colony. Furthermore, the viscous properties of agar make it challenging to decipher the mechanics of colony expansion as applied forces will dissipate over time. Polyacrylamide gels, however, are linearly elastic with negligible viscous dissipation and linearly deform in response to a wide range of stress. Polyacrylamide gels can be covalently linked with proteins or molecules of interest and chemical ligands to the surface to study the integrated effects of specific surface chemistry and substrate mechanics on cell behavior [Fig. 1(c)].^25^

In this manuscript, we experimentally investigate the effects of substrate stiffness and surface chemistry on the colony expansion in *M. xanthus*. We use polyacrylamide hydrogels with tunable mechanical properties and surface adhesion. We find a range of stiffness over which colony expansion rates increase with increasing substrate stiffness. Using *M. xanthus* mutants deficient in social or adventurous motility, we show that this effect depends strongly on the adventurous motility mode. Coating surfaces with chitosan promotes flare formation and changes the magnitude of expansion but does not change the overall trends with substrate stiffness. Our results underscore the importance of extracellular physical cues on the collective expansion of social bacteria, which has implications for understanding possible mechanosensing strategies utilized by the bacterium.

## RESULTS

### Design and characterization of PAA hydrogels

In the study, we are using polyacrylamide (PAA) gels of varying substrate stiffness and made a comparison with commonly used agar gels. Polyacrylamide gels are biologically inert and have well-defined linear elastic properties, making them ideal substrates for studying the effects of extracellular matrix stiffness on cell behavior. We varied the polyacrylamide concentration from 4% to 12% and characterized their mechanical properties by performing oscillatory rheology, as we described before.^28^ supplementary material Table 1 shows representative mechanical data of PAA and agar gels. The data present the shear storage modulus G′, which characterizes the elastic response of the material, and the loss modulus G″, which captures viscous dissipation and the fluid-like component of the material. The rheology of a 2% agar substrate has a significant G″ value (approximately 6 kPa) of the same order of magnitude as its G′ value (approximately 8 kPa). In contrast, the PAA gel is linearly elastic with mechanical response dominated by its elastic storage modulus (G′ ≫ G″). In some cases, we vary G′ of the PAA gels by increasing the amount of bis-crosslinker while holding the acrylamide concentration constant at 8% (Methods) to probe how changes in the pore size of the matrix impact the colony response Figs. 3(b) and 3(c).^28^ The data in supplementary material Fig. 1(b) demonstrates one of the advantages of PAA gels for understanding how bacteria respond to variations in substrate stiffness, by allowing us to focus on conditions in which the substrate yields a linearly elastic response and diminishes the effect of complex viscoelastic material properties.

### Substrate stiffness increases M. xanthus colony expansion rates

Our experimental protocol consists of observing the growth of *M. xanthus* colonies on the surface of hydrogel substrates with time-lapse microscopy (Methods). Before inoculating, the PAA gels are soaked multiple times in CTTYE nutrient-rich broth, and we deposit a small inoculum of bacteria on the gel surfaces (Fig. 1).

**FIG. 2.**
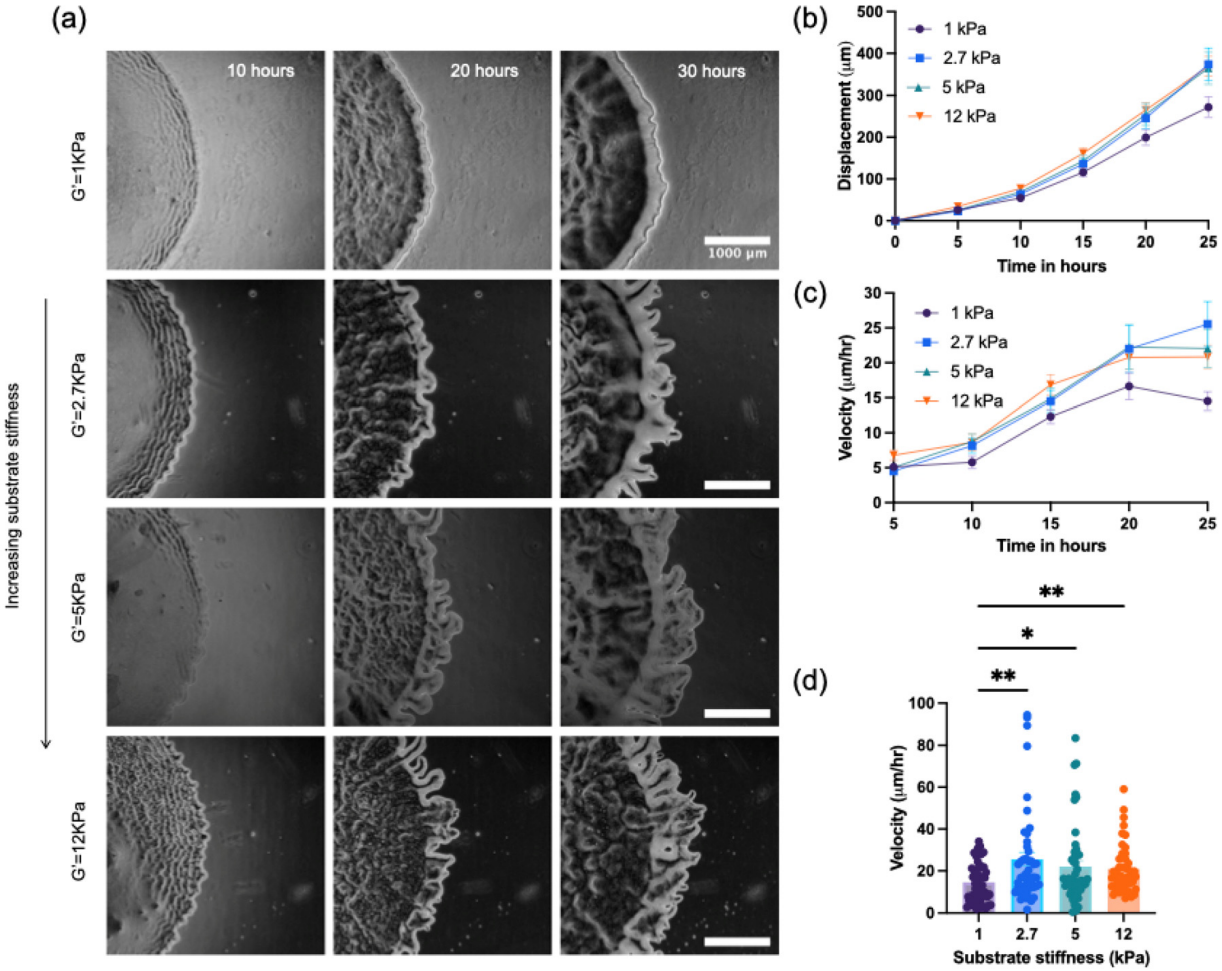
*M. xanthus* wild type colony expansion on PAA surfaces of varying stiffness. Qualitative and quantitative comparison of colony edge expansion phenotype on PAA substrates of varying stiffness. (a) Representative images of qualitative differences in colony edge expansion phenotypes of *M. xanthus* DK1622 (WT) on substrates of increasing (top to bottom) stiffness at 10, 20, and 30 h after inoculation. (b) Colony edge displacement with time, showing greater colony expansion on stiffer PAA gels. (c) Velocity of expanding colony edge over time and comparison of colony edge expansion velocity between the (d) 20 and 25 h timepoint. (Scale bar: 1 mm).

**FIG. 3.**
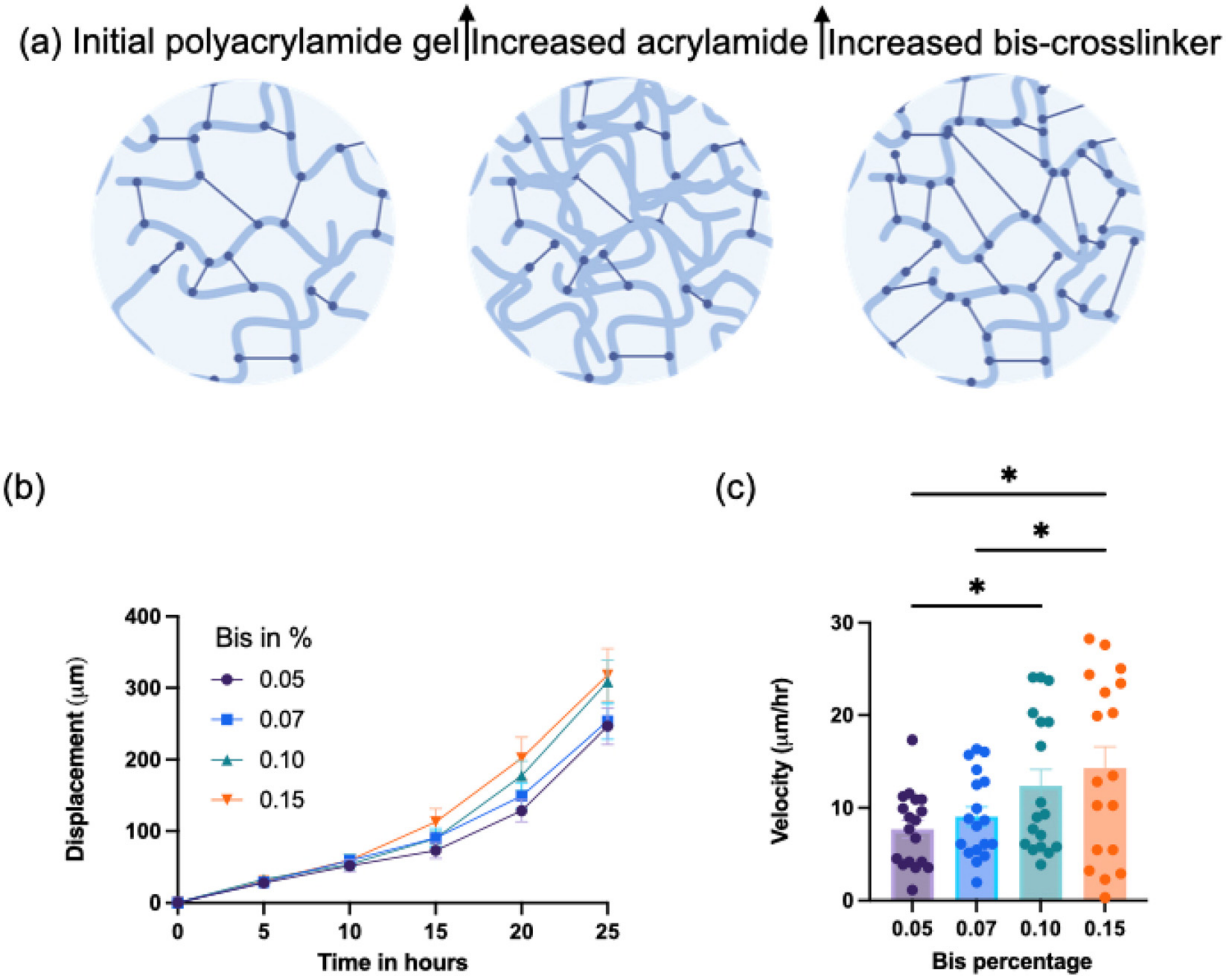
*M. xanthus* wild type colony expansion on PAA surfaces of increasing bis-crosslinker. (a) Schematic illustration of the effects of increasing acrylamide content vs bis-crosslinker on the hydrogel properties. Both effects increase the hydrogel stiffness, but the impact of bis-crosslinker on the hydrogel mesh/pore size is significantly less when compared to increasing acrylamide. (b) Velocity of expansion colony edge over time (c) The colony expansion rate increases with increasing substrate stiffness (1.5–5 kPa) via bis-crosslinker percentage (0.05–0.15) with constant acrylamide percentage in the gel mixture. Velocity measured at 10–20-h timepoint.

Representative images of the *M. xanthus* colony expansion are shown in Fig. 2(a). The images show differences in the shape of the colony edge and the colony expansion rates with changes in PAA concentration. In particular, we find prominent protrusions from the colony edge on stiffer PAA gels (5–12 kPa), while the protrusions and edge fluctuations are strongly suppressed on the softer PAA gels (1 kPa). To quantify the colony expansion, we measure the position of the furthest flair representative of the mean colony expansion at every 5-h interval over 25 h and compute the mean colony expansion velocity as a function of PAA concentration (supplementary material Fig. 2). The data show an increase in colony expansion rates with increasing PAA concentration. This enhancement with PAA concentration is somewhat counterintuitive since *M. xanthus* colonies are known to have slower expansion rates on more concentrated (and stiffer) agar substrates. Indeed, for this same species, the colonies are smaller on more concentrated agar^8,23^ (supplementary material Fig. 3). We also note that the velocity of the expanding edge colony is smaller compared to that on agar substrates, which we attribute to the smaller pore size in the PAA gels compared to agar that could impact the diffusion of nutrients to the cells on the substrate^28,29^ and differences in cell-substrate adhesion that we will explore further here.

Similar increases in colony expansion rates with increasing PAA concentration have been previously reported in studies focusing on the bacterium *Serratia marcescens.*^28^ It was argued that for *S. marcescens* increased colony expansion rates originated from a fluid dynamic model involving osmotic swelling of the colony, which has been described for several members of gamma proteobacteria (*E. coli* and *V. cholera*) and the gram-positive bacteria *B. subtilis.*^5,33–35^ In this model, as cells secrete components of the extracellular polymer matrix, an osmotic pressure gradient is generated between the colony and the underlying substrates, allowing fluid to flow from the substrate into the colony matrix resulting in increased colony growth. However, *M. xanthus* colony expansion on solid surfaces is predominantly through the slime trails, A- and S-motility modes,^36^ and cell division.^18,37^ Unlike *S. marcescens* and *B. subtilis*, in which extracellular polymeric substances (EPS) play a predominate role in encasing the colony and promoting three-dimensional colony expansion, in *M. xanthus* the EPS facilitates the formation of the slime trails that cells use to expand out against a surface. Therefore, we expect new mechanisms may be responsible for substrate-dependent colony expansion beyond EPS-generated osmotic stresses mediating *M. xanthus* expansion, which we further explore here.

In addition to substrate stiffness, another physical feature of a hydrogel substrate is its pore size. The substrate pore size can impact the diffusion of nutrients from the substrate into the colony^38^ and has also been hypothesized to affect the density of the binding sites between the cell and the substrate.^6^ Increasing the amount of PAA not only increases the substrate stiffness but also impacts the pore size of the underlying substrate. To examine whether *M. xanthus* colonies were responsive toward changes in substrate stiffness or network pore size, we designed PAA gels of varying stiffness but similar pore size by varying the amount of bis-crosslinker and holding the concentration of PAA the same. We have previously estimated and characterized the pore size of these PAA gels via diffusion and Poisson ratio measurements.^28^ This shows how designing gels by increasing bis-acrylamide-crosslinker holds the pore size nearly constant within experimental error.

**FIG. 4.**
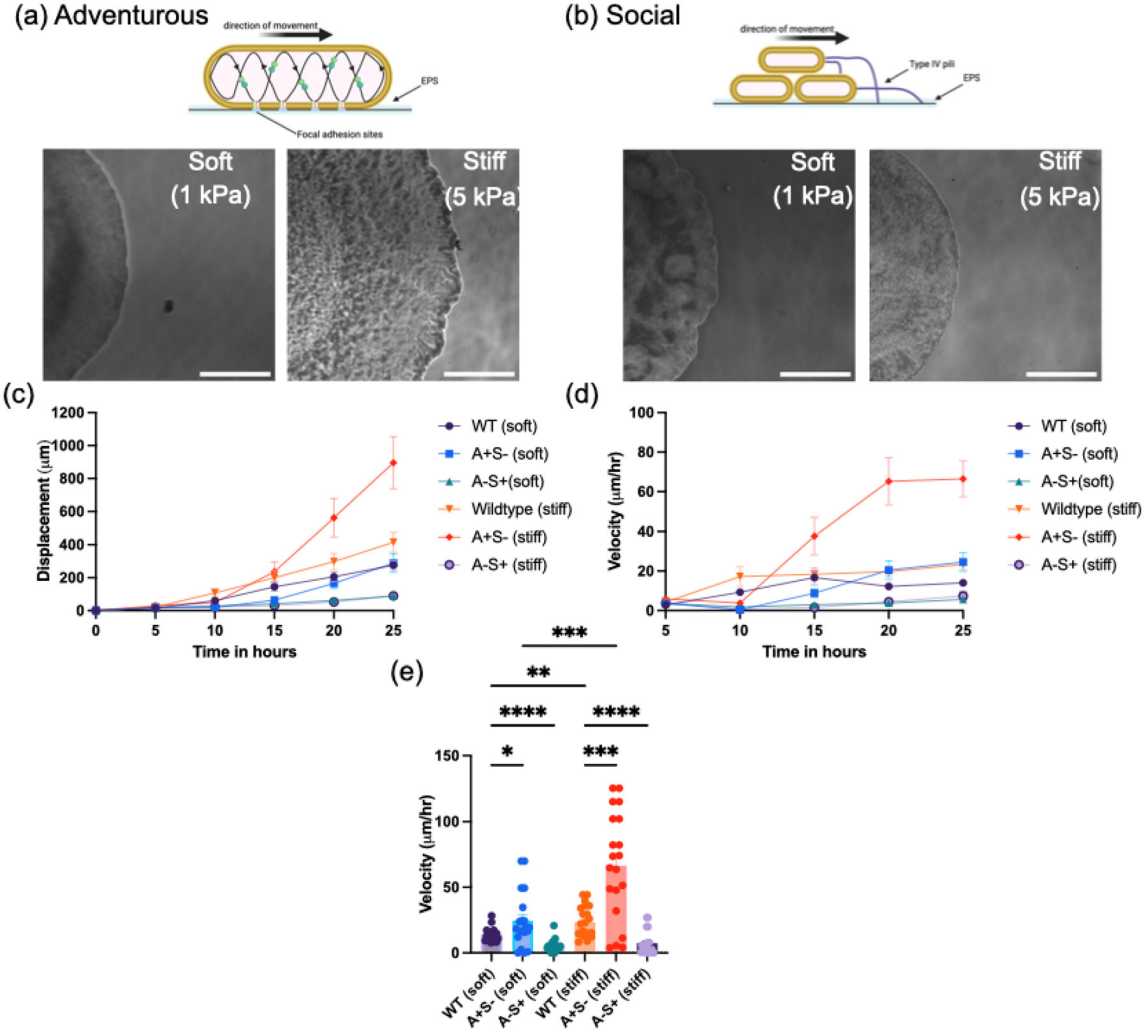
*M. xanthus* wild type and mutants' colony expansion on soft (1 kPa) and stiff (5 kPa) PAA substrates. Representative images showing the colony edge phenotypes of *M. xanthus* (a) DK1253 (A^+^S^−^) and (b) DK1218 (A^−^S^+^) on soft and stiff PAA substrates, respectively, 30 h after inoculation. Colony edge displacement with time on (c) soft (1 kPa) and stiff (5 kPa) PAA substrates alongside (d) velocity comparison of both. (e) Distribution of velocities at the 20–25 h timepoint. (Scale bar: 1 mm). (Scale bar: 1000 *μ*m).

Figure 3 shows the colony expansion rate as a function of bis-acrylamide-crosslinker, increasing from 0.05% to 0.15%, corresponding to G′ values from approximately 1.5 to 5 kPa.^28^ Figure 3(a) shows how changing the amount of acrylamide and bis-acrylamide changes the gel properties; an increase in acrylamide and bis-acrylamide concentration increases substrate stiffness, while increasing acrylamide concentration only impacts pore size, while increasing bis-acrylamide concentration maintains a constant pore size within experimental error (supplementary material Fig. 4). These results are strikingly similar to the effect of increasing substrate stiffness with increasing acrylamide chains namely, the colonies showed increased expansion rates on PAA gels of increasing bis-acrylamide-crosslinker, showing that in the limit of these purely elastic gels, *M. xanthus* is capable of responding to the stiffness (G′) of the substrate independently of the substrate network pore size.

### Effects of A- and S-motilities on substrate-dependent response

To connect the wild type colony expansion to *M. xanthus* motility modes, we next examined the expansion behavior of colonies in species defective in either A- or S-motility (Fig. 4). Here, we used two historic reference strains, DK1218 (*cglB2*), defective in A-motility (A^−^ S^+^), and DK1253 (*tgl-1*), defective in S-motility (A^+^ S^−^).^18,23^ Figure 4 shows representative images of A^−^ S^+^ and A^+^ S^−^ on soft (1 kPa) and stiff (5 kPa) polyacrylamide hydrogels. Most of the colonies show a rough boundary edge, although the flare-like structures seen in wild type are not observed. The A^−^ S^+^ colony on the 5 kPa gel differs in its colony edge shape, exhibiting a very smooth edge, consistent with the social mode of motility.

The colony edge displacement and velocity for wild type, A ^+^ S^−^, and A-S+ are shown in Figs. 4(c)–4(e). The data show that the expansion of A ^+^ S^−^ colonies significantly varies with substrate stiffness, increasing mean colony speeds from approximately 25 *μ*m/h on 1 kPa gels to approximately 70 *μ*m/h on 5 kPa gels. The A^–^S^+^ colonies, however, show no mean difference in colony expansion rates at a velocity of almost 6 *μ*m/h on both surfaces. Interestingly, on both soft gels (1 kPa) and stiff gels (5 kPa), we find that the colony expansion rate of the A ^+^ S^−^ is faster than wild type. Taken together, the data in Fig. 4 indicate that the adventurous motility mode of *M. xanthus* is sensitive to changes in substrate stiffness and is involved in increasing colony expansion rates on stiffer substrates.

### Substrate surface chemistry influences M. xanthus colony expansion

Our experimental data thus far show *M. xanthus* colonies expand at different rates on surfaces of different elastic moduli. Thus far, the colonies have been cultured on bare polyacrylamide hydrogels, which are generally considered inert to biological activity and chemical binding.^25^ To further investigate this interaction, we prepared PAA gels covalently linked with surface chemicals to these otherwise non-adhesive substrates (Methods). In particular, we coated the surfaces with chitosan, a natural N-Acetylglucosamine (GlcNAc) polymer, which *M. xanthus* putatively binds to. The specificity of this interaction was highlighted in studies showing the direct binding of pilin protein to chitosan required for pilus retraction.^39^ We also chose chitosan in this study as a possible starting point for a ligand-like candidate as *M. xanthus* has putative chitosan-binding proteins.^40^

**FIG. 5.**
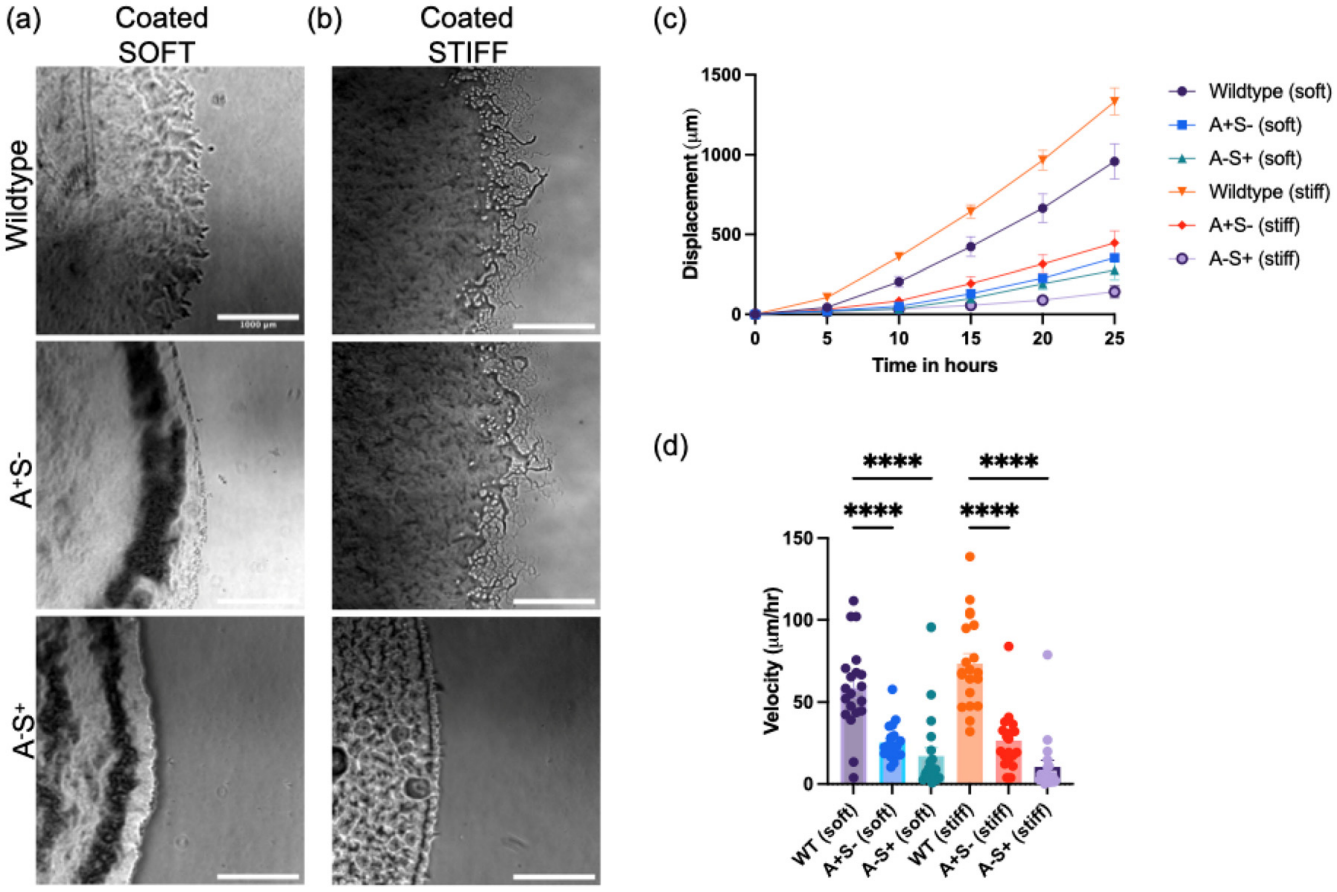
Effects of adhesive polymer chitosan surface presentation on colony expansion. Representative images of colony edge expansion phenotypes of *M. xanthus* DK1622 (WT), DK1253(A^+^S^−^), and DK1218(A^−^S^+^) on (a) soft (left) and (b) stiff (right) PAA gel substrates coated with chitosan (30 h past inoculation). (c) Colony edge displacement with time of DK1622 (WT), DK1253(A^+^S^−^), and DK1218(A^−^S^+^) on coated PAA gels. (d) Velocity comparison of DK1622 (WT), DK1253(A^+^S^−^), and DK1218(A^−^S^+^) on coated PAA gels (Scale bar: 1 mm).

Figures 5(a) and 5(b) show representative images of the wild type, A ^+^ S^−^, and A^−^S^+^ colonies on the chitosan-coated surfaces. On the chitosan-coated surfaces, prominent flairs associated with *M. xanthus* A-motility can be observed in wild type and A ^+^ S^−^ colonies on the stiff substrates, which suggests that flair formation may be substrate stiffness dependent. The colony expansion velocities are shown in Fig. 5(d). We observe differences in the trend of rate of expansion with increasing substrate stiffness when compared to the uncoated gels, namely, wild type colonies expand at faster rates on both substrates (as opposed to faster expansion rates on bare stiff substrates); A^−^S^+^ and A ^+^ S^−^ colonies have approximately the same average colony speed on both surfaces, and they undergo significantly slower expansion when compared to wild type (see supplementary material Fig. 5 for temporal velocity data). Another feature of coating the gels with chitosan is an increase in wild type colony expansion speeds compared to the uncoated surfaces (Fig. 4; see supplementary material Fig. 6 for direct comparison). Interestingly, the A ^+^ S^−^ cells, in contrast, show a decrease in expansion velocity on the chitosan-coated gels compared to uncoated gels.

One difference between the chitosan-coated and bare gels is the relative colony expansion rates between wild type and A ^+^ S^−^ cells. Namely, the A ^+^ S^−^ colonies expand slower on chitosan-coated surfaces when compared to wild type cells, whereas the opposite was observed on bare gels (SI Fig. 6).

Taken together, Fig. 5, shows that substrate stiffness does not significantly impact wild type *M. xanthus* and A ^+^ S^−^ expansion under conditions where the substrate is chemically modified to present an adhesive chemical (chitosan) on its surface but rather plays a role in enhancing expansion rates on both soft and stiff substrates, Figs. 5(d) and 5(e). Overall, the data in Figs. 4 and 5 (also, supplementary material Figs. 5 and 6) show that the collective colony expansion depends on both substrate stiffness and the presence of specific adhesive biomolecules on the substrate surface, consistent with mechanobiology studies in animal cells, and could arise from changes in binding and unbinding kinetics between cell surface proteins and the chitosan (and possibly even mechano-transduction pathways).

## DISCUSSION

Cell–matrix interactions are a central component of collective bacterial colony expansion. Bacterial colonies change their migration behavior on surfaces with different physical properties, but how they coordinate this process collectively remains largely unclear. Here, we examined the migration of the social bacterium *M. xanthus* on synthetic polyacrylamide hydrogels with tunable stiffness and surface chemistry. We found that *M. xanthus* colony expansion increases with substrate stiffness for uncoated polyacrylamide gels. Furthermore, using *M. xanthus* motility mutants, we found that the expansion rate of surface adhesion-based adventurous motility increased with substrate stiffness on uncoated substrates, whereas pili-based social motility was less responsive. Our results suggest that the surface adhesion-based adventurous motility mode of *M. xanthus* enables colonies to modify their expansion on different surfaces and is sensitive to changes in both substrate stiffness and surface chemistry.

This study was designed to understand better *M. xanthus* colony expansion by developing and extending mechanobiology techniques that were first pioneered in mammalian cell studies in the early 2000s.^41,42^ Cellular mechanobiology is a broad field that studies how mechanical forces impact biological materials. Although it began with relatively straightforward and well-designed experiments, the field has experienced substantial growth and advancement in experimental techniques. However, classically, three natural steps of progression demonstrate that cells can sense and respond to the mechanical features of their environment.

The first step is typically an experimental demonstration that cells exhibit different phenotypes on surfaces with different mechanical features. Linearly elastic materials, such as the PAA gels of varying stiffness used here, are commonly used for mammalian cells because of their tunable, well-defined mechanical properties and are optically opaque.^43,44^ There are a few studies with bacteria on these PAA gels, despite their usefulness in this field. This study shows that *M. xanthus* colonies modify expansion phenotypes in a stiffness-dependent manner. Furthermore, the effects of stiffness on a linear PAA gel are quite different from the effects generated by increasing agar stiffness by increasing agar concentration, which varies multiple other features of the agar substrate (e.g., pore size and viscoelasticity).

The second step is to address the mechanism, which can arise from different scenarios. In our case, we do not know *a priori* as to how the cells are responding. We can pose three general hypotheses as follows: (1) physical forces alone are changing the cell phenotype and cells are biologically behaving the same way (passive mechanisms), (2) biological activity changes on different surfaces through some cell-sensing mechanism (mechanosensing), and (3) some combination of the latter is occurring. Our data provide several important clues, which we discuss here.

Generally, cell phenotypes are a consequence of their environment and will vary as a function of substrate stiffness and adhesive linkages to the substrate surface. Bacteria are known for their remarkable ability to not only adhere to surfaces non-specifically but to also possess surface adhesive proteins that bind specific extracellular proteins. This motivated us to perform the experiments on bare, uncoated PAA gels vs covalently linked chitosan. We found that on bare surfaces, the colonies expanded more quickly on stiffer gels, whereas on coated surfaces, the expansion of wild type *M. xanthus* was enhanced on both soft and stiff substrates. This shows that the stiffness-dependent response of velocity with substrate stiffness is only dominant in bare conditions, and surface adhesion plays a role in enhancing expansion rates irrespective of substrate stiffness.

What is the effect of surface adhesion in our experiments? Our two experimental conditions suggest two different mechanisms. First, we show that the presence of *M. xanthus* binding protein (chitosan) increases both the presence of flairs and the rate of expansion of wild type cells. These are indicators of better adhesion between the cells and the surface and suggest that specific adhesion is at play between the cells and chitosan. Second, the mutant data point to the role of EPS slime deposition, as slime deposition in *M. xanthus*, is mediated by A-motility. On the uncoated gels, the greatest expansion rates are for the mutants with only A-motility, and the slowest expansion is for mutants lacking A-motility. These data also highlight the interplay between the two motility systems. Interestingly, chitosan-coating mediates which species expands fastest. Wild type is faster than mutants with only A-motility on chitosan-coated surfaces. We can speculate that some form of positive interplay between the two motility systems occurs when chitosan is present.

There are several avenues for future investigations. Future studies could consider broadening the number and concentration of EPS proteins covalently linked to the gel surfaces to identify the adhesive molecules that modify migration. We note *M. xanthus* have been hypothesized to have integrin-like adhesions^45^ as part of the A-motility gliding machinery, in part based on the identification of a von Willebrand A domain-containing outer-membrane protein CglB that couples the gliding transducer at the cell-substrate adhesion sites. We also note that the G′ values of the polyacrylamide hydrogels range from approximately 500 to 12 000 Pa (supplementary material Table I), as it is broad enough to show increases in colony speed with increasing stiffness. *M. xanthus* live primarily in soil, which has complex rheological properties^46,47^ and is also a 3D environment that future studies could consider in more detail. Finally, future studies could consider the influence of substrate stiffness on colony force generation and cellular transcriptional changes, which could demonstrate a possible mechano-sensing mechanism.

## CONCLUSION

Taken together, our results indicate a key role of substrate stiffness and adhesive coating in mediating the collective expansion of a social bacterium. Our data provide new evidence that the *M. xanthus* gliding machinery modifies colony response on different surfaces and may serve as a potential mechanosensing apparatus. The understanding gained here highlights the significance of chemo-mechanical interactions between bacteria and their surroundings as pivotal factors in colony expansion.

## METHODS

### Cell culture

There were three different *Myxococcus xanthus* strains used in this study*: M. xanthus* DK1622 (wild type), DK1253(A ^+^ S^−^), and DK1218(A^−^S^+^). To culture *M. xanthus*, cells were inoculated and grown in CTTYE medium with shaking at 32 °C overnight. Cell suspensions were then diluted to an absorption of 1.0 at OD600 in cell medium, and 5 *μ*l of inoculum was spotted on growth substrates. After inoculating on the substrate, cells were afterward maintained at 32 °C for *M. xanthus* for up to 17 h.

### Gel preparation

Polyacrylamide gels were prepared, as described previously,^28^ of varying stiffness with 4% (G′ = 1000 Pa), 6% (G′ = 2700 Pa), 8% (G′ = 5000 Pa), and 12% (G′ = 12 000 Pa) along with 0.15% bis-acrylamide. Polymerization was initiated by adding 1.6 *μ*l electrophoresis grade tetramethylethylenediamine (TEMED) followed by 4.8 *μ*l of 2% ammonium per-sulfate (APS) per 600 *μ*l of final gel solution. For each gel, a total of 200 *μ*l of the solution was then pipetted between two circular glass coverslips, one treated with glutaraldehyde (18 mm in diameter) (bottom) and the other SurfaSil-treated (22 mm in diameter) (bottom). The gels were then allowed to polymerize for 20 min. Once the gels were polymerized, the gel and coverslip arrangements were flipped and the SurfaSil-treated coverslip was removed from the gels. The final dimensions of the hydrogel formed a disk, approximately 18 mm in diameter and 0.8 mm in height. To prepare PAA substrates for inoculation, the protocol previously described by Tuson *et al.*^29^ was followed. The PAA gels were washed three times (quick wash, 10-min wash, and overnight wash) with TPM buffer. The washes were then repeated with CTTYE medium. Before inoculating, the substrates were removed from the growth medium and dried for 20 min with UV sterilization.

### Substrate coating

Two different types of coating were used for the gels, using protocols adopted from prior work.^48^ The gels were coated in two steps: (1) first, gels were coated with Sulfo-SANPAH [sulfosuccinimidyl 6–(4′-azido-2′nitrophenylamino) hexanoate]; Sulfo-SANPAH was mixed with DI water by vortexing in a 15 ml centrifuge tube. Then, 1 ml of the mixture was pipetted on top of each gel and Sulfo-SANPAH was allowed to activate by placing the gels under a UV lamp for 20 min. In the second step, gels were coated with chitosan. Chitosan for coating was prepared first by dissolving 10 mg chitosan in 3 ml of 0.2 M acetic acid, then diluted to a final ratio of 1:50 with DI water. Coatings were placed on gels and incubated at room temperature for 1 h. The gels were then washed with CTTYE following the same three-step wash.

### Rheological characterization

Rheology measurements were performed on a Malvern Panalytical Kinexus Ultra + rheometer with a 20 mm diameter plate. The elastic gel solutions were polymerized at room temperature between the rheometer plates at a gap height of 1 mm (30 min). The shear modulus was then measured as a function of shear strain from 5% to 50% at 1 radian/second frequency.

### Imaging

Time-lapse imaging was performed with a Nikon Ti-E inverted microscope equipped with a 4× objective and the Leica DMi8 inverted microscope equipped with a 4× objective. The cultures were maintained at 32 °C for *M. xanthus* using a Tokai-Hit stage top incubator. Phase contrast images were taken every 30 min for 30 h using a motorized stage to capture growth at two positions along the edge of each biofilm. For each colony, two videos were taken at different point locations of the colony edge.

### Statistical methods

Data in each plot represent at least 8 data points per experimental condition. Two colonies on two separate gels were analyzed in each experiment. For each colony, two videos were analyzed at different locations on the colony edge (yielding at least four measurements per condition per experiment). Each independent experiment was repeated on five different days at least. The Brown–Forsythe and Welch ANOVA test with 95% confidence level was used to determine statistical differences between distributions. Denotations: ^*^p ≤ 0.05; ^**^p ≤ 0.01; ^***^p ≤ 0.001; ^****^p ≤ 0.0001; and n.s., p > 0.05.

## SUPPLEMENTARY MATERIAL

See the supplementary material for additional datasets and supplementary figures and tables that support the findings discussed in the main text. These resources are intended to enhance the reader's understanding and provide further context to the research.

## Data Availability

The data that support the findings of this study are available from the corresponding author upon reasonable request.
